# Preterm Birth: Analysis of Longitudinal Data on Siblings Based on Random-Effects Logit Models

**DOI:** 10.3389/fpubh.2016.00278

**Published:** 2016-12-23

**Authors:** Silvia Bacci, Francesco Bartolucci, Liliana Minelli, Manuela Chiavarini

**Affiliations:** ^1^Department of Economics, University of Perugia, Perugia, Italy; ^2^Department of Experimental Medicine, University of Perugia, Perugia, Italy

**Keywords:** generalized linear mixed model, health care, Italy, maternal characteristics, standard certificate of live birth

## Abstract

**Introduction:**

The literature about the determinants of a preterm birth is still controversial. We approach the analysis of these determinants distinguishing between woman’s observable characteristics, which may change over time, and unobservable woman’s characteristics, which are time invariant and explain the dependence between the typology (normal or preterm) of consecutive births.

**Methods:**

We rely on a longitudinal dataset about 28,603 women who delivered for the first time in the period 2005–2013 in the Umbria Region (Italy). We consider singleton physiological pregnancies originating from natural conceptions with birthweight of at least 500 g and gestational age between 24 and 42 weeks; the overall number of deliveries is 34,224. The dataset is based on the Standard Certificates of Life Birth collected in the region in the same period. We estimate two types of logit model for the event that the birth is preterm. The first model is pooled and accounts for the information about possible previous preterm deliveries, including the lagged response among the covariates. The second model takes explicitly into account the longitudinal structure of data through the introduction of a random effect that summarizes all the (time invariant) unobservable characteristics of a woman affecting the probability of a preterm birth.

**Results:**

The estimated models provide evidence that the probability of a preterm birth depends on certain woman’s demographic and socioeconomic characteristics, other than on the previous history in terms of miscarriages and the baby’s gender. Besides, as the random-effects model fits significantly better than the pooled model with lagged response, we conclude for a spurious state dependence between repeated preterm deliveries.

**Conclusion:**

The proposed analysis represents a useful tool to detect profiles of women with a high risk of preterm delivery. Such profiles are detected taking into account observable woman’s demographic and socioeconomic characteristics as well as unobservable and time-constant characteristics, possibly related to the woman’s genetic makeup.

**Trial registration:**

Not applicable.

## 1 Introduction

The World Health Organization defines a Preterm Birth (PTB) as any birth before 37 completed weeks of gestation or 259 days since the first day of the woman’s last menstrual period. This is opposed to a full-term birth, which corresponds to any birth between 37 and 42 completed gestational weeks. Data on the incidence of PTB are very relevant in terms of public health: 9.0–11.0% of all births worldwide is preterm, with the highest rates of PTBs in Africa and North-America (11.9 and 10.9% of all births, respectively), whereas the lowest rates are observed in Europe (6.2%) (1, 2). A PTB can be classified into two groups: medically indicated PTB and idiopathic PTB. Medically indicated PTB derives from induction of labor or elective cesarean section before 37 completed weeks of gestation for maternal medical conditions (e.g., preeclampsia, diabetes), fetal problems, or other non-medical reasons, such that the risk for the fetus or the mother is superior to the benefit of continuing the pregnancy (3, 4). Idiopathic PTB may be regarded as a syndrome that, from a quiescence status, leads the uterus to active contractions before 37 completed gestational weeks.

Multiple causes may concur to a PTB, including infections or inflammations of urinary tract, vaginal infections, vascular diseases (e.g., hypertension, thrombophilia), diabetes, gestosis, polidramnios, and uterine overdistension. According to the literature, one of the most common causes of PTB is a maternal infective disease, even if the prevalence of a cause depends on several elements (5), among which gestational age, maternal age, and country-specific factors. More precisely, in developing countries, the main causes of PTB include infectious diseases and poor accessibility to and availability of health-care resources. In high-income countries, the increasing incidence of PTB is related to the increase of the average age of women at the moment of conception and the increasing use of fertility medicines that typically lead to multiple pregnancies. Nevertheless, both in rich and poor countries, a certain proportion of PTBs [up to half of all cases (6)] still lacks a precise explanation (1, 2).

A better understanding of the causes of PTB is a relevant subject of research in terms of public health. In fact, PTB is known to be a significant cause of child mortality and morbidity worldwide, and it is the leading cause of neonatal mortality: it is the direct cause of the 29% of deaths in children until 28 days and of the 11% until 5 years of age (7–9). In addition, PTB has lifelong effects on the development of the neural system, increasing the risk of cerebral palsy, impaired learning, and visual disorders, other than an increased risk of medical disabilities affecting the working capacity, such as obesity, diabetes, and hypertension (10, 11). Consequently, relevant economic costs derive from PTB in terms of neonatal intensive care, ongoing health care, educational needs, and social costs, as many families experience the sudden loss of a preterm baby or a stressful hospital stay lasting some months (2); see also Ref. (12) for detailed estimates of costs related to preterm children in England and Wales.

Our contribution is aimed at studying the main determinants of PTB in a cohort of 28,603 Italian women who delivered at least once and for the first time since January 1, 2005, until December 31, 2013, and who had singleton pregnancies originating from natural conceptions; the total size of babies/deliveries amounts to 34,224. For our purposes, anonymized routine data are used. These data come from merging the archives of certificates collected in the Umbria Region (Italy) in the period 2005–2013 and contain numerous information about woman’s and her partner’s characteristics, further to characteristics of each pregnancy and each newborn. It is important noting that the data at issue have a longitudinal structure with babies/deliveries nested within women. This allows us to analyze the effect of previous PTBs on the probability of a preterm delivery.

Our analysis is based on two types of logit model for the event that the birth is preterm. We initially fit a pooled regression model, which accounts for previous PTBs through the inclusion of the lagged response among the covariates. Then, we consider a random-effects logit model (13–15), which distinguishes from the pooled one for the introduction of a normally distributed individual effect summarizing all the unobservable characteristics of a woman affecting the probability of PTB across consecutive pregnancies. We aim at understanding whether there exists a true state dependence between consecutive preterm deliveries or if this dependence is spurious as only due to unobservable time-constant woman’s characteristics (16). We advise the reader that the present study is based on observational data and, therefore, it does not allow to conclude in terms of causal relations between response variable and covariates, but just in terms of statistically significant correlations. Naturally, the possible absence of significant correlation between the response and any covariate may suggest absence of such causal relations.

The proposed analysis is also relevant as it allows us to predict the probability of PTB, distinguishing between the first birth and the following ones. In the case of the first birth, the probability of PTB is predicted on the basis of the observed covariates and of a prior hypothesis about the random-effects distribution, whereas, for following births, previous history in terms of PTBs contributes to update the probability of another PTB.

## 2 Materials and Methods

### 2.1 Dataset Description

The study is based on data about a cohort of women who delivered in the Umbria Region (Italy) since January 1, 2005, until December 31, 2013. The study was carried out in compliance with the Italian law on personal privacy (Art. 20-21, D. L. 196/2003) and the regulations of the Regional Health Authorities about data management. Data were anonymized by the regional statistical office that also assigned to each patient a unique identifier within all administrative databases. Although the identification of the patient is impossible, this identifier allows us to merge information from different sources and to build a longitudinal dataset that includes information from different babies/deliveries of the same woman. When anonymized administrative data are used to inform health-care planning activities, the study is exempt from notification to the Ethics Committee and no specific written consent is needed to use patient information stored in the hospital databases.

In Italy, the State law requires that some information is collected for all births and, to ensure that a uniform methodology is applied in regional surveys and to obtain datasets containing comparable indicators, all regions are required to rely on the same form, which is known as Standard Certificate of Live Birth (SCLB). The midwife or obstetrician who attends the birth, or the doctor responsible for the delivery in the operation theater, must complete the SCLB within 10 days after the event. Each SCLB reports infant’s information such as gestational age, birthweight, gender, and date of birth. It also reports information regarding mother’s health status, socio-demographic characteristics (e.g., age, citizenship, educational attainment) and occupational status, number of previous miscarriages, type of conception (natural or assisted), obstetric procedures, characteristics and method of delivery (e.g., normal delivery or caesarian section), and inclusion of any abnormal conditions or congenital anomalies of the infant, cause of mortality, and information about use of prenatal care services (for further details, see Decree number 349/2001 of the Italian Ministry of Health). We remark that information on the detailed phenotype of PTB (i.e., medically indicated versus idiopathic preterm birth) are not available in the SCLB.

As already mentioned, for our study, we consider women who delivered at least once and for the first time in the period 2005–2013. We also consider just singleton births originating from natural conceptions and with a birthweight of at least 500 g and a gestational age between 24 and 42 (included) weeks (note that in Italy it is not possible to deliver after 42 weeks; after this term the labor is induced). Moreover, pregnancies with a physiological course are taken into account, being pathological a pregnancy for which some adverse conditions arise, such as autoimmune diseases, multiple pregnancies, hypertension, presence of pelvic mass, Rh isoimmunization, or diabetes. In other words, exclusion criteria are multiple births, pregnancies originating from assisted reproduction methods, and pregnancies characterized by a pathological course, which represent well-known risk factors for PTB (17–19). Therefore, their inclusion in these factors could obscure the significant effect of other determinants. Moreover, the decision to consider deliveries with a gestational age of at least 24 weeks is driven by the medical practice that considers this as the boundary between miscarriage and PTB, whereas the value of 500 g for the birthweight represents the minimum threshold to consider a fetus capable of an independent life. Overall, the longitudinal dataset we analyze comprises 28,603 women and 34,224 babies/deliveries. The 81.37% (23,274) of these women delivered just once in the period at issue, whereas the 17.65% (5,049) of women delivered twice and the remaining 0.98% of women gave birth to three (268) or four (12) infants in the period we consider.

### 2.2 Variables of Interest

Table 1 reports the list of variables taken into account in the study, together with some basic descriptive statistics. Some variables, such as some partner’s characteristics, are ignored in the study due to missing data; birthweight is also ignored as it is an outcome of pregnancy occurring in conjunction with gestational age [a recent study that relates birthweight to gestational age is illustrated in Ref. (20)]. We specify that, having dropped variables with missing data, the remaining variables used in the study do not present problems of unobserved values.

**Table 1:** **Description and distribution of variables**.

Variable description	Category	Proportion
PTB	Preterm birth (<37 weeks)	0.036
	Full-term birth	0.964
Woman’s age	<20 years	0.021
	20–34 years	0.762
	≥35 years	0.217
Woman’s citizenship	Italian	0.787
	Other Western	0.006
	East-European	0.132
	Other	0.075
Woman’s education	Compulsory diploma	0.191
	High school diploma	0.512
	Degree or higher	0.297
Woman’s occupational status	No job	0.324
	Employee	0.542
	Freelancer or manager	0.134
Partner’s occupational status	No job	0.041
	Employee	0.670
	Freelancer or manager	0.289
Previous miscarriages	None	0.853
	1 miscarriage	0.120
	≥2 miscarriages	0.026
Parity	First baby	0.836
	Pluriparous	0.164
Interval between pregnancies (*only pluriparous*)	<6 months	0.028
	6–12 months	0.123
	12–18 months	0.148
	≥18 months	0.702
First medical check	≤3 months	0.981
	>3 months	0.019
Baby’s gender	Female	0.483
	Male	0.517

The response variable of interest, PTB, is binary and it is equal to 1 for a delivery happening before 37 gestational weeks and to 0 otherwise. We observe (Table 1) that preterm deliveries are the 3.6% of the total number. As previously illustrated, we do not account for high-risk pregnancies, and this value is lower than the overall Italian PTB rate (6.9%). Among pluriparous women, this percentage rises to 13.2% in the case of women who delivered preterm at the previous born, whereas it is equal to 2.3% for women who had a previous full-term birth.

Concerning the possible determinants of PTB, we observe that the great majority of women is between 20 and 34 years old (76.2%), is Italian (78.7%), has a job position (67.6%), mainly as employee (54.2%), and has an employee (67.0%) or a freelancer or manager partner (28.9%). Besides, around one-half of women has a high school diploma, whereas less than one-third has a degree or a higher level of education (e.g., master, PhD); the remaining 19.1% of women attained at most a compulsory qualification.

Regarding the childbearing history, the 85.3% of women did not have any miscarriage, and the 12.0% had just one miscarriage. Furthermore, the 83.6% of women is waiting her first born and, among those who are waiting a following baby, the 70.2% conceived more than 18 months after the previous delivery, whereas the time interval between a pregnancy and the following conception is between 12 and 18 months in the 14.8% of cases and between 6 and 12 months in the 12.3% of cases. We also observe that the first prenatal visit usually happens within the end of the first trimester (98.1%). Finally, among the newborns 48.3% are females, and 51.7% are males.

### 2.3 Statistical Method

The longitudinal structure of the available data, due to deliveries nested within women, allows us to account for the dependence between the type of delivery (preterm or not) for different pregnancies of the same mother. To properly account for such a dependence, we adopt two types of logit model for the event that the birth is preterm.

Let *i* denote a woman in the database, with *i* = 1, …, *n*, and, for each woman, let *j* denote a baby/delivery, with *j* = 1, …, *J_i_*. Moreover, let *y_ij_* be the binary response variable equal to 1 if delivery *j* is preterm (PTB) and to 0 otherwise (full-term birth). We also introduce a vector of covariates, *x_ij_*, collecting observed woman’s characteristics, which may change with pregnancy, and observed baby/pregnancy characteristics. In particular, vector *x_ij_* collects the variables listed in Table 1, that is, the lagged response, woman’s age, woman’s citizenship, woman’s education, woman’s and her partner occupational status, previous miscarriages, parity, time interval between a pregnancy and the following conception, gestational age for the first medical check, and baby’s gender.

The first model we use for the analysis is a pooled logit model based on the assumption

(1) $$\text{log}\frac{p(y_{ij}=1|x_{ij})}{p(y_{ij}=0|x_{ij})}=\beta_{0}+{x\prime }_{ij}^{\;}\beta ,\;i=1,\;\ldots ,\;n,\;j=1,\;\ldots ,\;J_{i},$$

where *β*_0_ is the constant term and *β* is the column vector of regression parameters. This model is characterized by the inclusion, among the covariates, of the lagged response variable. In such a way, we account for the information from previous deliveries in terms of gestational age and, then, for the longitudinal structure of the data. The main drawback of this naive approach is that it does not explicitly consider the effect on the probability of PTB of unobserved (and unobservable) woman’s characteristics, which are time constant and tend to affect the probability of PTB across repeated pregnancies. This is an additional effect with respect to that of the observed and, usually time-varying, covariates. Therefore, we also estimate a random-effects logit model (13–15, 21), which originates from model (1) by introducing a latent component *u_i_*, specific of each woman, as follows:

(2) $$\text{log}\frac{p(y_{ij}=1|u_{i},x_{ij})}{p(y_{ij}=0|u_{i},x_{ij})}=\beta_{0}+{x\prime }_{ij}^{\;}\beta +u_{i},\;i=1,\;\ldots ,\;n,\;j=1,\;\ldots ,\;J_{i}.$$

As usual, the random effects *u_i_* are assumed to be independent and normally distributed with mean equal to 0 and constant variance $\sigma_{u}^{2}$. In practice, each *u_i_* summarizes all the mother’s unobservable time-constant characteristics and captures the unobserved heterogeneity between women in terms of risk of having a preterm delivery. A possible interpretation is that this random effect represents the effect on PTB of the genetic characteristics of the woman it is referred to. We outline that, in principle, vector *x_ij_* in equation (2) includes the lagged PTB response variable, similarly to equation (1). We expect that, in the presence of a spurious correlation between outcomes of subsequent births in terms of gestational ages, the regression coefficient of the lagged response is significant in the pooled model (1), but not in the random-effects model (2).

From equation (2), it is clear that the probability of PTB, that is,

(3) $$p(y_{ij}=1|u_{i},x_{ij})=\frac{1}{1+exp\left[-\left(\beta_{0}+{x\prime }_{ij}^{\;}\beta +u_{i}\right)\right]},\;i=1,\;\ldots ,\;n,\;j=1,\;\ldots ,\;J_{i},$$

depends both on the observed values of the covariates in *x_ij_* and on the value assumed by the random component *u_i_* for the *i*-th woman: values of *u_i_* much higher (smaller) than zero imply a higher (smaller) probability of PTB with respect to an “average” woman, being constant all the observed covariates. Therefore, if we are interested in using model (3) for predictive purposes (13, 22), we may consider two scenarios:

• for a woman at her first birth, the best prediction of probability of PTB is obtained by substituting in equation (3) the observed values of covariates in *x_ij_* and value 0 in *u_i_*;
• for a pluriparous woman, we may use the woman’s history in terms of previous events of preterm deliveries to update the value of *u_i_*, which will generally differ from 0. More in detail, we expect a posterior value of *u_i_* greater than 0 in the case of women who had at least a previous PTB and a posterior value of *u_i_* less than zero in the case of women who never experienced a PTB. In practice, for a woman at her second baby, we compute the empirical Bayes estimates of random effects (13) on the basis of the estimates of regression parameters *β*_0_ and *β* and, then, we calculate their expected value conditionally on *y_i_*_1_ = 1 and to *y_i_*_1_ = 0: the results provide the posterior predicted values of *u_i_* if the first birth took place preterm and if the first birth took place full term, respectively. In the case of a third or a successive pregnancy, the expected values of the random effects are obtained along the same lines, taking into account the outcomes of all the previous pregnancies.

Rather than replacing *u_i_* with a point estimate as described above, a procedure that provides rather similar results consists in computing the predicted probability of PTB by integrating equation (3) on the prior distribution of random effects, in the case of a primiparous woman, and on the posterior distribution, in the case of a pluriparous woman.

The two proposed models, based on assumptions (1) and (2), are compared in terms of goodness-of-fit through certain well-known information criteria, which are commonly used for the choice between non-nested models (i.e., models which cannot be obtained one from the other through suitable constraints on the parameters): the Akaike’s Information Criterion [AIC (23)] and the Bayesian Information Criterion [BIC (24)]. Both criteria are based on a penalized version of the log-likelihood function, so as to account for the model complexity (measured by the number of free parameters) and, in the case of the BIC index, for the sample size.

The first criterion is based on estimating the Kullback–Leibler divergence between the true distribution and the estimated distribution of the data and accounts for the tradeoff between goodness-of-fit (measured by the log-likelihood) and complexity (in terms of number of parameters) of the model. It is defined on the basis of the following index

$$AIC=-2\hat{\ell }+2\;\#\text{par},$$

where $\hat{\ell }$ is the maximum log-likelihood for the model of interest and #par denotes the number of free parameters. The BIC criterion relies, instead, on a larger penalty for the model complexity than AIC, being based on the following index

$$BIC=-2\hat{\ell }+\text{log}(n)\;\#\text{par},$$

where *n* is the sample size, that is, the number of women considered in the study.

According to the criteria described above, one should prefer the model with the smallest value of AIC or BIC. Another useful index to assess the random-effects model is based on the Intraclass Correlation Coefficient [ICC (21)], which corresponds to the proportion of variance of the response variable due to woman’s unobservable characteristics accounted by the mother-specific latent effect component, that is,

$$ICC=\frac{\sigma_{u}^{2}}{\sigma_{u}^{2}+\frac{\pi^{2}}{3}},$$

where π^2^/3 represents the variance of the latent effect at baby/delivery level, having a standard logistic distribution, and, therefore, $\sigma_{u}^{2}+\frac{\pi^{2}}{3}$ is the total variance of *y_ij_* [for more details see, among others, Ref. (21)].

## 3 Results and Discussion

Tables 2 and 3 report the estimates of the regression coefficients in the pooled logit model (model M0) and the random-effects logit model (model M1), respectively. In both cases, the odds ratios are shown with the corresponding SEs and limits of the confidence intervals (at 95% level) denoted by *l*_1_ and *l*_2_, other than the value of the test statistic and the corresponding *p*-value; for the random-effects model we also report the estimate of the ICC.

**Table 2:** **Estimates of demographic and socioeconomic characteristics for PTB: pooled logit model (model M0)**.

Covariate	Category	Odds ratio	SE	*z*-Value	*p*-Value	*l*_1_	*l*_2_
Constant		0.020	0.003	−28.440	<0.001	0.016	0.027
Previous PTB	Full-term birth	–	–	–	–	–	–
	Preterm birth	6.345	1.543	7.600	<0.001	3.940	10.218
Woman’s age	20–34 years	–	–	–	–	–	–
	<20 years	1.068	0.194	0.370	0.715	0.749	1.524
	≥35 years	1.258	0.091	3.170	0.002	1.092	1.449
Woman’s citizenship	Italian	–	–	–	–	–	–
	Other Western	1.054	0.383	0.150	0.884	0.517	2.148
	East-European	1.177	0.105	1.830	0.068	0.988	1.401
	Other	1.089	0.122	0.760	0.449	0.874	1.357
Woman’s education	Compulsory diploma	–	–	–	–	–	–
	High school diploma	0.825	0.064	−2.490	0.013	0.710	0.960
	Degree or higher	0.773	0.069	−2.870	0.004	0.648	0.922
Woman’s occupation status	Employee	–	–	–	–	–	–
	No job	1.150	0.082	1.950	0.051	0.999	1.322
	Freelancer or manager	1.074	0.099	0.770	0.440	0.896	1.287
Partner’s occupation status	Employee	–	–	–	–	–	–
	No job	1.250	0.164	1.700	0.089	0.966	1.618
	Freelancer or manager	1.018	0.069	0.260	0.793	0.892	1.162
Previous miscarriages	None	–	–	–	–	–	–
	1 miscarriage	1.060	0.095	0.650	0.514	0.890	1.262
	≥2 miscarriages	1.524	0.234	2.740	0.006	1.127	2.059
Parity	Second baby or more	–	–	–	–	–	–
	First baby	1.720	0.191	4.890	<0.001	1.384	2.137
Interval between pregnancies	≥18 months	–	–	–	–	–	–
	12–18 months	0.983	0.245	−0.070	0.945	0.604	1.601
	6–12 months	0.823	0.226	−0.710	0.478	0.481	1.409
	<6 months	1.285	0.559	0.580	0.564	0.548	3.015
First medical check	≤3 months	–	–	–	–	–	–
	>3 months	1.292	0.236	1.410	0.160	0.904	1.848
Baby’s gender	Female	–	–	–	–	–	–
	Male	1.222	0.072	3.420	0.001	1.090	1.371

According to the results in Table 2, we note that the odds ratio of having a preterm delivery is significantly affected by the woman’s history in terms of previous PTB (*p*-value < 0.001): the odds ratio for delivering before 37 gestational weeks is more than 6 times (limits of confidence interval at 95%: *l*_1_ = 3.94 and *l*_2_ = 10.22) higher for pluriparous women who already experienced a PTB with respect to pluriparous women who experienced a previous full-term delivery. A recent study (25) confirmed that multiple previous spontaneous PTBs and no previous preterm births are independent risk factors for recurrence. In particular, authors found that approximately one in six women with a previous spontaneous PTB suffered a recurrent spontaneous PTB.

We also observe a highly significant effect for the following characteristics: woman’s age, previous miscarriages, parity, and baby’s gender. More in detail, the odds of PTB are higher for primiparous women (odds ratio = 1.720; *l*_1_ = 1.384 and *l*_2_ = 2.137) older than 35 years (odds ratio = 1.258; *l*_1_ = 1.092 and *l*_2_ = 1.449), who are waiting a male infant (odds ratio = 1.222; *l*_1_ = 1.090 and *l*_2_ = 1.371) and experienced in their past at least two miscarriages (odds ratio = 1.524; *l*_1_ = 1.127 and *l*_2_ = 2.059). On the contrary, a mother’s age less than 20 years and the experience of only one miscarriage do not affect in a significant way the risk of preterm delivery.

According to certain studies, an extreme maternal age represents a risk factor for preterm deliveries (26–28), although Carmichael et al. (29) recently described a null effect of being very young white woman and a positive effect of being older white woman. Another crucial typology of risk factors for PTB is represented by the woman’s childbearing history, such as previous PTBs, multiple pregnancies, hypertensive disease of pregnancy, and other woman’s characteristics, such as a low maternal body-mass index, pre-existing non-communicable diseases, or infections (30–32). In this regard, our analysis outlines that women with previous histories of miscarriages tend to have a higher risk of PTB; see also Ref. (33) for a similar result.

In addition, neither the time of the first medical check (which can be considered as a proxy of the prenatal care) nor the time interval between a pregnancy and the following conception have any significant effect on the probability of PTB. This result is not in agreement with the literature (34) that demonstrated the association between a short period of time between birth and a subsequent conception with adverse perinatal outcomes such as preterm birth, even after adjusting for concomitant risk factors.

Among the covariates related to the socioeconomic status, we outline the relevance of the educational level. Women with a high school diploma (odds ratio = 0.825; *l*_1_ = 0.710 and *l*_2_ = 0.960; *p*-value = 0.013) and, more evidently, with a degree or another university title (odds ratio = 0.773; *l*_1_ = 0.648 and *l*_2_ = 0.922; *p*-value = 0.004) have a reduced risk of PTB with respect to women with just a compulsory diploma. More in detail, a low socioeconomic position is generally associated with an increased risk of PTB and, among socioeconomic measures, the woman’s educational level is the strongest predictor of PTB (35, 36). Generally speaking, several studies have shown that education is a valuable dimension of socioeconomic status, and it allows us to consistently predict the health status, especially for women and their children (37).

A moderately significant effect (*p*-value < 0.10) is also observed for the woman’s citizenship, with women from East-Europe disadvantaged with respect to the other ones, and for the occupational status, being the unemployment condition of the woman or of her partner an element of disadvantage with respect to the status of worker.

The previous results are in agreement with the main stream of the literature [for a recent systematic review, see Ref. (38)], according to which, in addition to the past and current pregnancy history and current stress factors, socioeconomic factors are associated with PTB, including social class, usually assessed by earnings and education, working condition (professional status, ergonomic environment, working hours), physical and traveling activities, daily life activities, lifestyle, family status, emotional distress, and psychosocial conditions. Other studies suggest that partner’s socioeconomic status might also have a significant association with birth outcomes in general (39, 40) and, coherently with our results, with PTB (41). However, it must to be noted that, even though both parents play an important role in the socioeconomic status of a family, few studies have considered the combined effects of paternal and maternal characteristics on PTB and that, traditionally, maternal influence is believed to be more important for birth outcomes than paternal influence (42).

Estimates of the parameters of the random-effects model are very similar to those of the corresponding parameters of the pooled model for all the covariates, with the only exception of the lagged response variable (compare Table 2 with Table 3). After having controlled for the unobserved woman’s characteristics summarized through the random effects, the estimated value of the odds ratio for previous PTB is strongly reduced and is not significant, with a *p*-value equal to 0.617. This result suggests a spurious state dependence (16) between outcomes referred to consecutive deliveries. In other words, the association found by the pooled model (model M0) between gestational terms of consecutive deliveries is not due to a true state dependence between the involved variables (in the sense that the term of a given delivery affects the term of the following delivery for the same woman), but to the omission of time-constant woman’s characteristics affecting the probability of PTB at all pregnancies. On the contrary, a situation of true state dependence would correspond to estimated odds ratio and *p*-value substantially unchanged after including random effects to control for the unobserved and time-persistent woman’s characteristics taking also into account the longitudinal structure of the dataset.

**Table 3:** **Estimates of demographic and socioeconomic characteristics for PTB: logit model with random-effects and lagged PTB (model M1)**.

Covariate	Category	Odds ratio	SE	*z*-Value	*p*-Value	*l*_1_	*l*_2_
**Fixed effects**							
Constant		0.006	0.003	−10.560	<0.001	0.002	0.015
Previous PTB	Full-term birth	–	–	–	–	–	–
	Preterm birth	0.695	0.505	−0.500	0.617	0.167	2.891
Woman’s age	20–34 years	–	–	–	–	–	–
	<20 years	1.084	0.245	0.360	0.722	0.696	1.687
	≥35 years	1.321	0.121	3.040	0.002	1.104	1.580
Woman’s citizenship	Italian	–	–	–	–	–	–
	Other Western	1.062	0.478	0.130	0.893	0.440	2.565
	East-European	1.227	0.138	1.820	0.069	0.984	1.530
	Other	1.109	0.155	0.740	0.462	0.842	1.459
Woman’s education	Compulsory diploma	–	–	–	–	–	–
	High school diploma	0.789	0.077	−2.430	0.015	0.651	0.955
	Degree or higher	0.728	0.083	−2.780	0.005	0.581	0.911
Woman’s occupational status	Employee	–	–	–	–	–	–
	No job	1.201	0.107	2.050	0.040	1.008	1.431
	Freelancer or manager	1.093	0.123	0.790	0.431	0.876	1.363
Partner’s occupational status	Employee	–	–	–	–	–	–
	No job	1.315	0.217	1.660	0.098	0.951	1.819
	Freelancer or manager	1.026	0.085	0.310	0.753	0.873	1.207
Previous miscarriages	None	–	–	–	–	–	–
	1 miscarriage	1.062	0.116	0.550	0.580	0.858	1.316
	≥2 miscarriage	1.683	0.337	2.600	0.009	1.136	2.493
Parity	Second baby or more	–	–	–	–	–	–
	First baby	1.499	0.219	2.770	0.006	1.125	1.995
Interval between pregnancies	≥18 months	–	–	–	–	–	–
	12–18 months	0.979	0.277	−0.070	0.942	0.562	1.706
	6–12 months	0.814	0.253	−0.660	0.509	0.443	1.498
	<6 months	1.446	0.732	0.730	0.466	0.536	3.898
First medical check	≤3 months	–	–	–	–	–	–
	>3 months	1.377	0.316	1.400	0.163	0.879	2.158
Baby’s gender	Female	–	–	–	–	–	–
	Male	1.258	0.091	3.190	0.001	1.093	1.450
**Random effects**							
ICC		0.514	0.111			0.306	0.716

We also consider two variants of model M1 that are formulated to control for potential endogeneity due to the simultaneous presence of lagged PTB and random effects. First, we specify the linear predictor of model M1 conditionally on the covariates observed at the first delivery, and second, we add to the original formulation of model M1 the average values of time-varying covariates for each woman (43). In both cases, results (not shown here) confirm the absence of dependence between consecutive deliveries in terms of preterm births.

Values of AIC and BIC indices are shown in Table 4 for models M0, M1, and, additionally, for a random-effects model without the lagged response among the covariates (model M2), whose parameter estimates are shown in Table 5.

**Table 4:** **Goodness-of-fit for the estimated models (M0, M1, M2): estimated maximum log-likelihood value, number of parameters (# par), AIC, and BIC indices**.

Model	Random effects	Lagged PTB	Log-likelihood	# par	AIC	BIC
M0	No	Yes	−5248.4413	21	10538.883	10712.369
M1	Yes	Yes	−5246.5318	22	10537.064	10718.811
M2	Yes	No	−5246.6082	21	10535.216	10708.703

**Table 5:** **Estimates of demographic and socioeconomic characteristics for PTB: logit model with random-effects and without lagged PTB (model M2)**.

Covariate	Category	Odds ratio	SE	*z*-Value	*p*-Value	*l*_1_	*l*_2_
**Fixed effects**							
Constant		0.007	0.002	−18.330	<0.001	0.004	0.012
Woman’s age	20–34 years	–	–	–	–	–	–
	<20 years	1.080	0.233	0.360	0.722	0.707	1.650
	≥35 years	1.307	0.111	3.140	0.002	1.105	1.544
Woman’s citizenship	Italian	–	–	–	–	–	–
	Other Western	1.060	0.456	0.130	0.893	0.456	2.462
	East-European	1.216	0.129	1.840	0.065	0.988	1.498
	Other	1.104	0.148	0.740	0.459	0.849	1.435
Woman’s education	Compulsory diploma	–	–	–	–	–	–
	High school diploma	0.797	0.073	−2.480	0.013	0.666	0.953
	Degree or higher	0.738	0.079	−2.860	0.004	0.599	0.909
Woman’s occupational status	Employee	–	–	–	–	–	–
	No job	1.190	0.100	2.070	0.038	1.010	1.403
	Freelancer or manager	1.089	0.118	0.790	0.430	0.881	1.346
Partner’s occupational status	Employee	–	–	–	–	–	–
	No job	1.301	0.204	1.680	0.094	0.956	1.770
	Freelancer or manager	1.024	0.081	0.300	0.763	0.877	1.196
Previous miscarriages	None	–	–	–	–	–	–
	1 miscarriage	1.062	0.111	0.570	0.566	0.865	1.304
	≥2 miscarriages	1.648	0.309	2.660	0.008	1.141	2.380
Parity	Second baby or more	–	–	–	–	–	–
	First baby	1.558	0.183	3.770	<0.001	1.237	1.961
Interval between pregnancies	≥18 months	–	–	–	–	–	–
	12–18 months	0.980	0.271	−0.070	0.943	0.570	1.685
	6–12 months	0.814	0.248	−0.680	0.498	0.448	1.477
	<6 months	1.422	0.700	0.720	0.475	0.542	3.733
First medical check	≤3 months	–	–	–	–	–	–
	>3 months	1.359	0.297	1.400	0.160	0.886	2.084
Baby’s gender	Female	–	–	–	–	–	–
	Male	1.250	0.085	3.270	0.001	1.094	1.429
**Random effects**							
ICC		0.456	0.053			0.356	0.560

Both information criteria (Table 4) indicate that the best compromise between goodness-of-fit and complexity is provided by model M2, corroborating the above interpretation about the presence of unobserved factors affecting the gestational terms of consecutive deliveries of the same woman. Concerning the other covariates, parameter estimates of model M2 (Table 5) are coherent with those of the other models and, additionally, the selected model allows us to explain the 45.6% of the total variance of the probability of PTB.

**Figure 1:**
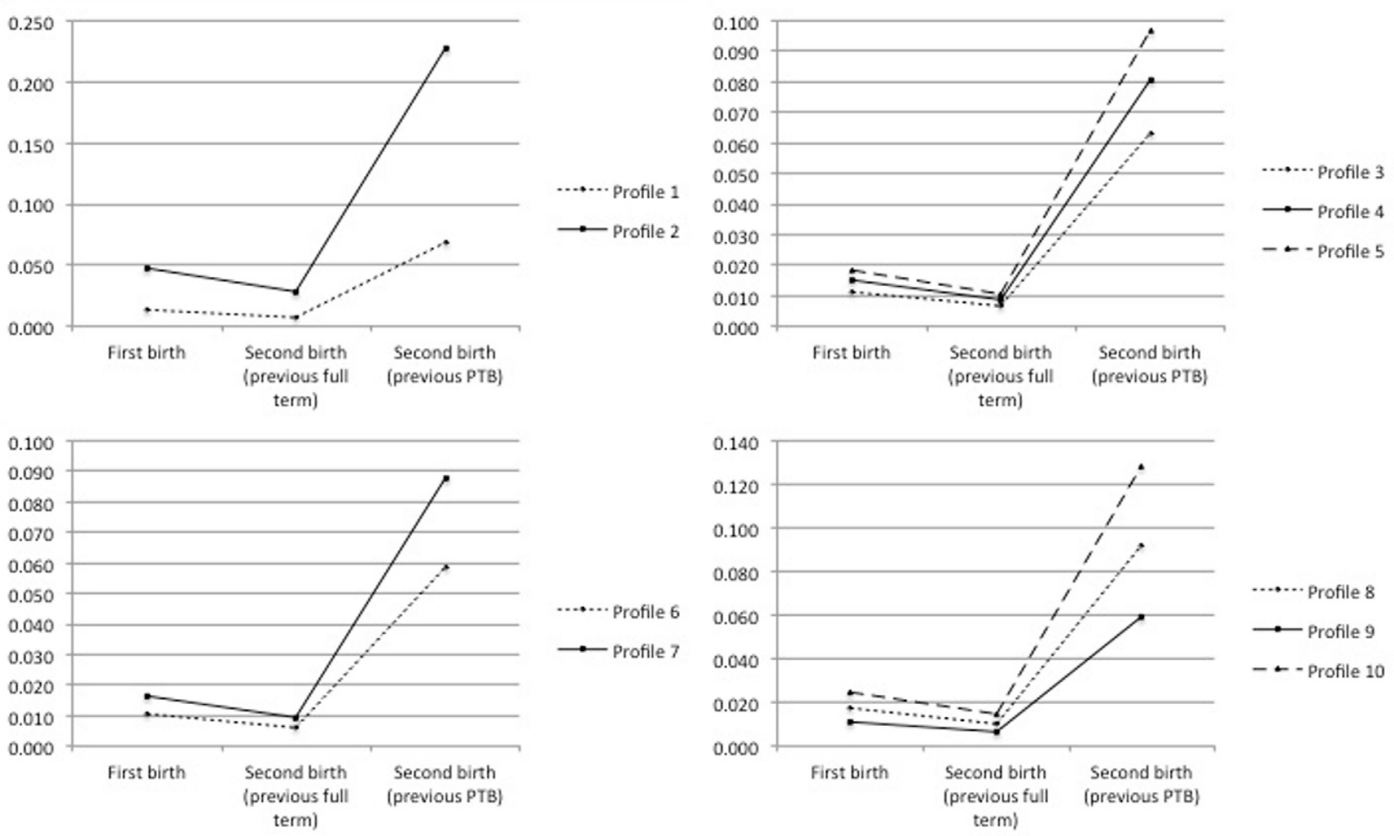
**Probability of PTB in the case of first birth, second birth given a previous full-term birth, and second birth given a previous PTB, for each profile defined in Table 6**.

Finally, we illustrate how the selected random-effects model M2 may be used for predictive purposes. As an example, in Table 6, some individual profiles are reported (row #1 of the table refers to a generic pregnant woman); for each of them the probability of PTB is calculated, distinguishing between primiparous and pluriparous women (in the specific case, women at her second delivery) and, related to this latter type of woman, between women who experienced a previous full-term birth and women who experienced a previous PTB. See also related Figure 1 for a graphical representation.

**Table 6:** **Estimated probabilities of PTB for some individual profiles, distinguishing between first births and second births**.

#	Woman’s age	Woman’s citizenship	Woman’s education	Woman’s occup. st.	Partner’s occup. st.	Previous miscar.	Baby’s gender	First birth	Second birth	
									Previous full term	Previous PTB
1	–	–	–	–	–	–	–	0.013	0.007	0.069
2	≥35	East-Europ.	Compul.	No job	No job	≥2	Male	0.047	0.028	0.227
3	20–34	Italian	–	–	–	–	–	0.012	0.007	0.063
4	≥35	Italian	–	–	–	–	–	0.015	0.009	0.081
5	≥35	East-Europ.	–	–	–	–	–	0.018	0.011	0.097
6	20–34	Italian	–	Employee	Employee	–	–	0.011	0.006	0.058
7	20–34	Italian	–	No job	No job	–	–	0.016	0.009	0.087
8	–	East-Europ.	Compul.	No job	Employee	–	–	0.017	0.010	0.092
9	20–34	–	Degree	–	–	–	–	0.011	0.006	0.059
10	≥35	–	–	–	–	≥2	–	0.025	0.014	0.128

The probabilities previously reported are predicted on the basis of the estimated regression coefficients of model M2. Besides, in the case of a first birth, we assume a value of the random effect equal to 0 (i.e., equal to the average value at population level), whereas in the case of a successive birth we update this value on the basis of the information about the gestational terms of the previous delivery. In this way, the expected probability of PTB for a generic primiparous woman (Table 6, row # 1; Figure 1, top left panel) equals 1.3%, whereas it reduces to 0.7% for a pluriparous woman whose previous pregnancy ended at full term and rises to 6.9% in the case of a previous preterm delivery. We also observe that these three expected probabilities rise to 4.7, 2.8, and 22.7%, respectively, in presence of particularly adverse conditions (Table 6, row # 2; Figure 1, top left panel). Similar conclusions are found by Kazemier et al. (44) who compare the risk of spontaneous PTB after a previous PTB and after a previous full-term pregnancy: they find that 22.1% of all singleton preterm births in the second pregnancy comes from women with a previous preterm singleton birth and that women with a previous singleton PTB have a statistically significant increased risk for recurrent PTB compared with women with a previous full-term birth (odds ratio 5.43, *l*_1_ = 4.03; *l*_2_ = 7.31).

## 4 Conclusions

Excluding cases affected by known reasons for PTB, such as pathological and multiple pregnancies as well as iatrogenic factors (e.g., intensive use of infertility treatments leading to increased rates of multiple pregnancies), the present study evaluates the state of reproductive science relevant to understanding the causes of PTB. The study also identifies potential targets for prevention and outlines challenges and opportunities for translating research findings into effective interventions.

The mechanisms underlying the process leading to a preterm delivery involve a wide range of biological, environmental, behavioral, social, and clinical factors and, with the exception of some known risk factors above mentioned, they remain poorly understood (45). In our study, we find that the outcomes of subsequent deliveries in terms of gestational age (preterm versus full term) are not correlated each other, after having controlled for time-persistent unobservable woman characteristics. This important conclusion suggests the existence of a sort of woman-specific predisposition responsible for repeated preterm deliveries and encourages future studies on the human genome; for conclusions in the same direction see also Ref. (46, 47).

The proposed study has also relevant policy implications, as it allows us to predict the probability of PTB for different individual profiles, distinguishing between primiparous and pluriparous women, and, related to this latter type of woman, between women who experienced a previous full-term birth and women who experienced a previous PTB. We outline that the estimation of individual probability of PTB is an essential process for patient-centered care (48). Indeed, individual risk-based counseling may motivate patients to learn about methods of risk reduction and to interpret early signs of preterm labor, and it may facilitate the decision-making process for preventive interventions. In this regard, we also find that the first medical check has no effect on PTB. This result agrees with the Cochrane systematic review (49) that evaluates the possible effects of a range of interventions on PTB. Few interventions have shown to be effective (e.g., cerclage, prophylactic progesterone, and fetal fibronectin testing), whereas for around a half of the interventions evaluated, the Cochrane review concluded that there is insufficient evidence to provide sound recommendations for clinical practice. Among interventions without a significant effect on PTB risk, there were risk scoring systems for predicting PTB, but even psychosocial interventions and alternative models of care, such as additional support versus usual care during at-risk pregnancy and specialized antenatal care versus routine care in singleton pregnancies at high risk of PTB.

Among the main strengths of our study, we recall that it is population based (and not sample based); therefore, the background characteristics of individuals do not differ at all from those of the general population. Besides, the study involves multiple factors that affect pregnancy and birth outcomes, accounting in an explicit way for the longitudinal structure of the data in which repeated pregnancies are nested within women. With respect to the state-of-art, we also stress the role of demographic and socioeconomic characteristics.

In conclusion, our study contributes to the existing literature in two main directions. First, the spurious correlation between subsequent deliveries suggests the existence of a time-persistent woman-specific predisposition responsible for repeated preterm deliveries, which could be attributable to genetic characteristics, so encouraging future studies on the humane genome. Second, spotting profiles of women who differ for the probability of having a preterm delivery allows us to identify potential target groups for prevention and outlines challenges and opportunities for translating research findings into effective interventions. On the other hand, we are aware of some limitations of the study, mainly related to the poor quality of some registered data (e.g., partner’s characteristics) deriving from possible inaccuracy of medical staff and related to the absence of some potentially relevant clinical information, such as diseases in pregnancy, distinction between idiopathic and medically indicated PTB, and smoking and drinking habits.

## Ethics Statement

No ethics approval and consent are necessary for participation, as data are collected by each pregnant woman who gives birth in Italy according to the Italian Government Law. *Consent for Publication*: Not applicable. *Availability of Data and Materials*: Data are available upon request by the Public Health Department of the Umbria Region (Italy).

## Author Contributions

The present work is the result of the ideas and the contribution of all the co-authors. SB and FB were mainly involved in the drafting of section Methods, whereas MC and LM mainly worked on sections Introduction and Conclusions. All authors equally contributed in the section concerning Results and Discussion.

## Conflict of Interest Statement

The authors declare that the research was conducted in the absence of any commercial or financial relationships that could be construed as a potential conflict of interest.

## Abbreviations

PTB, preterm birth; SCLB, Standard Certificate of Life Birth; AIC, Akaike’s information criterion; BIC, Bayesian information criterion; ICC, intraclass correlation coefficient.

## References

[1] Beck S Wojdyla D Say L Betran AP Merialdi M Requejo JH The worldwide incidence of preterm birth: a systematic review of maternal mortality and morbidity. Bull World Health Organ (2010) 88:31–8. 10.2471/BLT.08.062554 20428351 PMC2802437

[2] March of Dimes, PMNCH, Save the Children, and WHO. Born Too Soon. The Global Action Report for Preterm Birth. Geneva: World Health Organization (2012).

[3] Moutquin JM. Classification and heterogeneity of preterm birth. BJOG (2003) 110:30–3. 10.1046/j.1471-0528.2003.00021.x 12763108

[4] Goldenberg RL Gravett MG Iams J Papageorghiou AT Waller SA Kramer M The preterm birth syndrome: issues to consider in creating a classification system. Am J Obstet Gynecol (2012) 206:113–8. 10.1016/j.ajog.2011.10.865 22177186

[5] Steer P. The epidemiology of preterm labour. BJOG (2005) 112:1–3. 10.1111/j.1471-0528.2005.00575.x 15715585

[6] Menon R. Spontaneous preterm birth, a clinical dilemma: etiologic, pathophysiologic and genetic heterogeneities and racial disparity. Acta Obstet Gynecol Scand (2008) 87:590–600. 10.1080/00016340802005126 18568457

[7] Lawn JE Cousens S Zupan J Lancet Neonatal Survival Steering Team. 4 million neonatal deaths: when? Where? Why? Lancet (2005) 365:891–900. 10.1016/S0140-6736(05)71048-5 15752534

[8] Ezzati M Hoorn SV Lopez AD Danaei G Rodgers A Mathers CD Comparative quantification of mortality and burden of disease attributable to selected risk factors. In: Lopez AD Mathers CD Ezzati M Jamison DT Murray CJL, editors. Global Burden of Disease and Risk Factors. Washington, DC: The World Bank (2006). p. 241–68. 21250375

[9] Lozano R Naghavi M Foreman K Lim S Shibuya K Aboyans V Global and regional mortality from 235 causes of death for 20 age groups in 1990 and 2010: a systematic analysis for the global burden of disease study 2010. Lancet (2012) 380:2095–128. 10.1016/S0140-6736(12)61728-0 23245604 PMC10790329

[10] Moster D Lie R Markestad T. Long-term medical and social consequences of preterm birth. N Engl J Med (2008) 359:262–73. 10.1056/NEJMoa0706475 18635431

[11] Alexander BT Suttira I Preterm birth: a novel risk factor for higher blood pressure in later life. Hypertension (2012) 59:189–90. 10.1161/HYPERTENSIONAHA.111.186551 22158644

[12] Mangham LJ Petrou S Doyle LW Draper ES Marlow N. The cost of preterm birth throughout childhood in england and wales. Pediatrics (2009) 123:e312–27. 10.1542/peds.2008-1827 19171583

[13] Skrondal A Rabe-Hesketh S Generalized Latent Variable Modeling: Multilevel, Longitudinal, and Structural Equation Models. Boca Raton, FL: Chapman & Hall/CRC Press (2004).

[14] McCulloch CE Searle SR Neuhaus JM Generalized, Linear, and Mixed Models. Hoboken, NJ: Wiley (2008).

[15] Stroup WW Generalized Linear Mixed Models: Modern Concepts, Methods and Applications. Boca Raton, FL: Chapman & Hall/CRC Press (2012).

[16] Heckman JJ Heterogeneity and state dependence. In: Rosen S, editor. Studies in Labor Markets. Chicago: University of Chicago Press (1981). p. 91–139.

[17] Blickstein I. Does assisted reproduction technology, per se, increase the risk of preterm birth? BJOG (2006) 113:68–71. 10.1111/j.1471-0528.2006.01126.x 17206968

[18] Schaaf JM Mol BW bu Hanna A Ravelli AC. Trends in preterm birth: singleton and multiple pregnancies in the Netherlands, 2000-2007. BJOG (2011) 118:1196–204. 10.1111/j.1471-0528.2011.03010.x 21668771

[19] Qin J Wang H Sheng X Liang D Tan H Xia J. Pregnancy-related complications and adverse pregnancy outcomes in multiple pregnancies resulting from assisted reproductive technology: a meta-analysis of cohort studies. Fertil Steril (2015) 103:1492–508. 10.1016/j.fertnstert.2015.03.018 25910567

[20] Bacci S Bartolucci F Chiavarini M Minelli L Pieroni L. Differences in birthweight outcomes: a longitudinal study based on siblings. Int J Environ Res Public Health (2014) 11:6472–84. 10.3390/ijerph110606472 25003169 PMC4076673

[21] Snijders TAB Bosker RJ Multilevel Analysis. An Introduction to Basic and Advanced Multilevel Modeling. London: SAGE (2012).

[22] Skrondal A Rabe-Hesketh S Prediction in multilevel generalized linear models. J R Statist Soc A (2009) 172(3):659–87. 10.1111/j.1467-985X.2009.00587.x

[23] Akaike H Information theory and an extension of the maximum likelihood principle. In: Petrov BN Caski F, editors. Second International Symposium of Information Theory. Budapest: Akademiai Kiado (1973). p. 267–81.

[24] Schwarz GE Estimating the dimension of a model. Ann Stat (1978) 6:461–4. 10.1214/aos/1176344136

[25] Yamashita M Hayashi S Endo M Okuno K Fukui O Mimura K Incidence and risk factors for recurrent spontaneous preterm birth: a retrospective cohort study in Japan. J Obstet Gynaecol Res (2015) 41:1708–14. 10.1111/jog.12786 26311118

[26] Fraser AM Brockert JE Ward RH. Association of young maternal age with adverse reproductive outcomes. N Engl J Med (1995) 332:1113–8. 10.1056/NEJM199504273321701 7700283

[27] Chiavarini M Bartolucci F Gili A Pieroni L Minelli L. Effects of individual and social factors on preterm birth and low birth weight: empirical evidence from regional data in Italy. Int J Public Health (2012) 57:261–8. 10.1007/s00038-011-0311-3 22009490

[28] Caroland M Maternal age ≥45 years and maternal and perinatal outcomes: a review of the evidence. Midwifery (2013) 29:479–89. 10.1016/j.midw.2012.04.001 23159159

[29] Carmichael SL Cullen MR Mayo JA Gould JB Loftus P Stevenson DK Population-level correlates of preterm delivery among black and white women in the U.S. Midwifery (2014) 9:e94153. 10.1371/journal.pone.0094153 24740117 PMC3989227

[30] Goldenberg RL Culhane JF Iams JD Romero R Epidemiology and causes of preterm birth. Lancet (2008) 371:75–84. 10.1016/S0140-6736(08)60074-4 18177778 PMC7134569

[31] Plunkett J Muglia LJ. Genetic contributions to preterm birth: implications from epidemiological and genetic association studies. Ann Med (2008) 40:167–95. 10.1080/07853890701806181 18382883

[32] Muglia LJ Katz M The enigma of spontaneous preterm birth. N Engl J Med (2010) 362:529–35. 10.1056/NEJMra0904308 20147718

[33] Passini R Cecatti JG Lajos GJ Tedesco RP Nomura ML Dias TD Brazilian multi centre study of preterm birth (emip): prevalence and factors associated with spontaneous preterm birth. PLoS One (2014) 9:e109069 10.1371/journal.pone.0109069 25299699 PMC4192080

[34] DeFranco EA Stamilio DM Boslaugh SE Gross GA Muglia LJ. A short interpregnancy interval is a risk factor for preterm birth and its recurrence. Am J Obstet Gynecol (2007) 197:.e1–6. 10.1016/j.ajog.2007.06.042 17826413

[35] Koupilova I Vagero D Leon DA Pikhart H Prikazsky V Holcik J Social variation in size at birth and preterm delivery in the Czech Republic and Sweden, 1989-1991. Paediatr Perinat Epidemiol (1998) 12:7–24. 10.1111/j.1365-3016.1998.00075.x 9483614

[36] Morgen C Bjø rk C Andersen PK Mortensen LH Nybo Andersen AM Socioeconomic position and the risk of preterm birth: a study within the danish national birth cohort. Int J Epidemiol (2008) 37:1109–20. 10.1093/ije/dyn112 18577529

[37] Bloomberg L Meyers J Braverman MT. The importance of social interaction: a new perspective on social epidemiology, social risk factors, and health. Health Educ Q (1994) 21:447–63. 10.1177/109019819402100407 7843977

[38] Blumenshine P Egerter S Barclay CJ Cubbin C Braveman PA. Socioeconomic disparities in adverse birth outcomes: a systematic review. Am J Prev Med (2010) 39:263–72. 10.1016/j.amepre.2010.05.012 20709259

[39] Parker JD Schoendorf KC Kiely JL. Associations between measures of socioeconomic status and low birth weight, small for gestational age, and premature delivery in the United States. Ann Epidemiol (1994) 4:271–8. 10.1016/1047-2797(94)90082-5 7921316

[40] Messer LC Vinikoor LC Laraia BA Kaufman JS Eyster J Holzman C Socioeconomic domains and associations with preterm birth. Soc Sci Med (2008) 67:1247–50. 10.1016/j.socscimed.2008.06.009 18640759

[41] Shah P Knowledge Synthesis Group on Determinants of Preterm/Low Birthweight Births. Paternal factors and low birthweight, preterm, and small for gestational age births: a systematic review. Am J Obstet Gynecol (2010) 202:103–23. 10.1016/j.ajog.2009.08.026 20113689

[42] Chen X-K Wen SW Krewski D Fleming N Yang Q Walker MC Paternal age and adverse birth outcomes: teenager or 40+, who is at risk? Hum Reprod (2008) 23:1290–6. 10.1093/humrep/dem403 18256111

[43] Rabe-Hesketh S Skrondal A Multilevel and Longitudinal Modeling Using Stata. College Station, TX: Stata Press (2012).

[44] Kazemier BM Buijs PE Mignini L Limpens J de Groot CJ Mol BW. Impact of obstetric history on the risk of spontaneous preterm birth in singleton and multiple pregnancies: a systematic review. BJOG (2014) 121(10):1197–208. 10.1111/1471-0528.12896 24899245

[45] Chang H Larson J Blencowe H Spong CY Howson CP Cairns-Smith SL Preventing preterm births: analysis of trends and potential reductions with interventions in 39 countries with very high human development index. Lancet (2013) 381:223–34. 10.1016/S0140-6736(12)61856-X 23158883 PMC3572865

[46] Pennell CE Jacobsson B Williams SM Buus RM Muglia LJ Dolan SM Genetic epidemiologic studies of preterm birth: guidelines for research. Am J Obstet Gynecol (2007) 196:107–18. 10.1016/j.ajog.2006.03.109 17306646

[47] Jelliffe-Pawlowski LL Baer RJ Blumenfeld YJ Ryckman KK O’Brodovich HM Gould JB Maternal characteristics and mid-pregnancy serum biomarkers as risk factors for subtypes of preterm birth. BJOG (2015) 122:1484–93. 10.1111/1471-0528.13495 26111589 PMC4704442

[48] Iams JD Berghella V. Care for women with prior preterm birth. Am J Obstet Gynecol (2010) 203:89–100. 10.1016/j.ajog.2010.02.004 20417491 PMC3648852

[49] Piso B Zechmeister-Koss I Winkler R. Antenatal interventions to reduce preterm birth: an overview of cochrane systematic reviews. BMC Res Notes (2014) 7:265. 10.1186/1756-0500-7-265 24758148 PMC4021758

